# Nicotinamide Riboside—The Current State of Research and Therapeutic Uses

**DOI:** 10.3390/nu12061616

**Published:** 2020-05-31

**Authors:** Mario Mehmel, Nina Jovanović, Urs Spitz

**Affiliations:** 1 Biosynth Carbosynth, Rietlistrasse 4, 9422 Staad, Switzerland; m.mehmel@gmx.de; 2 Faculty of Biology, Department of Biochemistry and Molecular Biology, Institute of Physiology and Biochemistry, University of Belgrade, Studentski Trg 1, 11000 Belgrade, Serbia; ninajovanovic2202@gmail.com; 3 Biosynth Carbosynth, Axis House, High Street, Compton, Berkshire RG20 6NL, UK

**Keywords:** nicotinamide riboside, nicotinamide adenine dinucleotide, supplementation, safety, bioavailability, metabolic disorders, age-associated diseases, COVID-19

## Abstract

Nicotinamide riboside (NR) has recently become one of the most studied nicotinamide adenine dinucleotide (NAD^+^) precursors, due to its numerous potential health benefits mediated via elevated NAD^+^ content in the body. NAD^+^ is an essential coenzyme that plays important roles in various metabolic pathways and increasing its overall content has been confirmed as a valuable strategy for treating a wide variety of pathophysiological conditions. Accumulating evidence on NRs’ health benefits has validated its efficiency across numerous animal and human studies for the treatment of a number of cardiovascular, neurodegenerative, and metabolic disorders. As the prevalence and morbidity of these conditions increases in modern society, the great necessity has arisen for a rapid translation of NR to therapeutic use and further establishment of its availability as a nutritional supplement. Here, we summarize currently available data on NR effects on metabolism, and several neurodegenerative and cardiovascular disorders, through to its application as a treatment for specific pathophysiological conditions. In addition, we have reviewed newly published research on the application of NR as a potential therapy against infections with several pathogens, including SARS-CoV-2. Additionally, to support rapid NR translation to therapeutics, the challenges related to its bioavailability and safety are addressed, together with the advantages of NR to other NAD^+^ precursors.

## 1. Introduction

**Figure 1:**
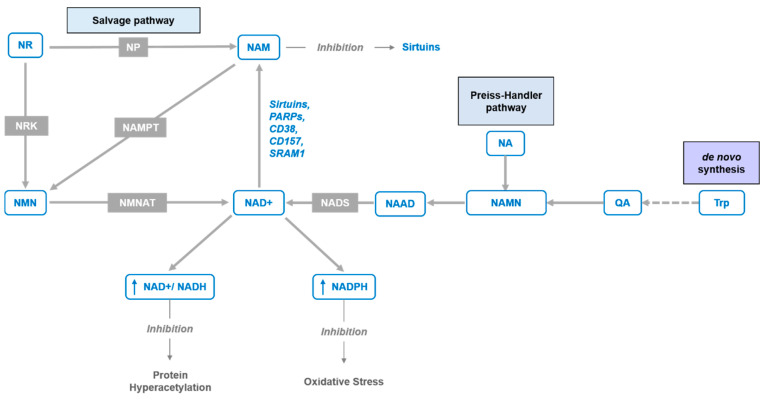
NAD^+^ synthesis pathways. The figure depicts NAD^+^ de novo pathway from tryptophan (Trp) through quinolinic acid (QA), Preiss–Handler pathway from nicotinic acid (NA) via nicotinic acid adenine dinucleotide (NAAD) and NAD synthetase (NADS), and “salvage pathways” from nicotinamide riboside (NR) and nicotinamide mononucleotide (NMN) via purine nucleoside phosphorylase (NP) and nicotinamide phosphoribosyltransferase (NAMPT) enzymes or nicotinamide ribose kinases (NRK) and NMN/NaMN adenylyltransferases (NMNAT), respectively.

**Figure 2:**
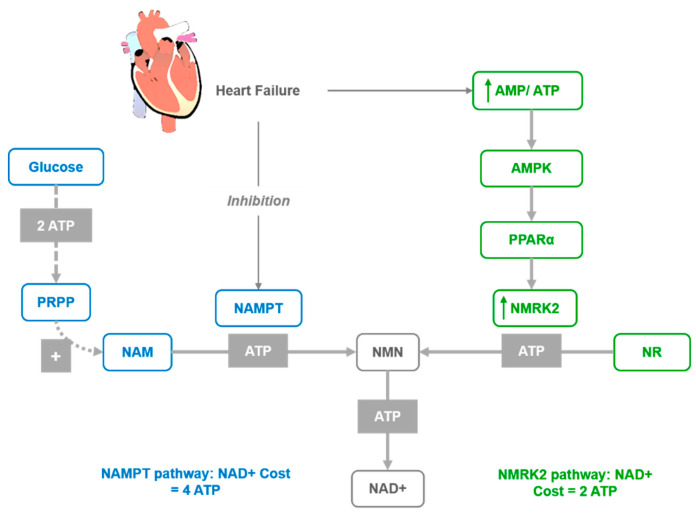
Activation of the NMRK2 pathway represent a common adaptive mechanism in the failing heart where NAD^+^ levels are low. NAD^+^ synthesis from NR through the NMRK2 pathway may be favored, as the NMN synthesis from NR by NMRK enzymes requires only one ATP molecule while synthesis from NAM by NAMPT requires at least three ATP equivalents.

In recent years, interest in NAD^+^ biology has been gaining momentum, revealing critical insights into its roles in numerous physiological processes and underlining the beneficial effects of supplementation with its precursors. Moreover, accumulating evidence indicates that a decrease in NAD^+^ levels contributes to the development of age-associated pathophysiology [1,2,3]. The systemic NAD^+^ decrease is caused by both lowered rates of biosynthesis and increased use of NAD^+^. The immense demand for NAD^+^ is caused by its importance in cellular oxidation–reduction reactions, including the majority of catabolic and anabolic reactions, such as glycolysis, fatty acid β-oxidation, tricarboxylic acid cycle, synthesis of fatty acids, cholesterol, steroids, etc. [4,5,6]. Additionally, NAD^+^-consuming enzymes, such as sirtuins, poly-ADP-ribose polymerases (PARPs), cADP-ribose synthases (CD38/157 ectoenzymes) [7,8,9] and mono-ADP-ribose transferases (ARTs) contribute to an overall depletion of NAD^+^. Biosynthesis can compensate somewhat to the depleted levels of NAD^+^ via de novo synthesis from tryptophan (Trp) or in the salvage pathways from four other precursors, nicotinamide (NAM), nicotinic acid (NA), nicotinamide riboside, and nicotinamide mononucleotide (NMN). While de novo synthesis from Trp is carried out in an eight-step pathway, the salvageable precursors NA and NAM require only three (Preiss–Handler pathway) and two steps, respectively (Figure 1). Nicotinamide riboside (NR) is an additional salvageable NAD^+^ precursor with a two-step [10] or three-step pathway [11] to form NAD^+^ (Figure 1). In mammals, the most common precursor is NAM, which can further be used to form NMN by the rate-limiting enzyme, phosphoribosyltransferase (NAMPT) [12]. In the final step, NMN is converted to NAD^+^ by NMN/NaMN adenylyltransferases (NMNATs) [13,14]. As a function of the aging process and/or overnutrition, NAD^+^ content and NAMPT expression are found to decline in multiple tissues [3,15,16,17], while the maintenance of NAD^+^ levels relies on diverse biosynthetic routes and precursors in different tissues [18,19]. Nevertheless, the decreased expression of NAMPT enzyme is one of the major causes of the NAD^+^ decline over age [15,20]. The requirement of this enzyme can be bypassed with the direct conversion of NR to NMN by two nicotinamide ribose kinases, NMRK1 and NMRK2 (also known as NRK1 and NRK2) [10]. This also circumvents the requirement of energetically costly PRPP (phosphoribosyl pyrophosphate. Figure 2) and the feedback inhibition by NAD^+^ [21]. Alternatively, NR can be turned into NAM by purine nucleoside phosphorylase (NP), which is subsequently converted to NAD^+^ via NMN by NMNAT (Figure 1). Hence, the utilization of NR depends on the expression of either the Nrk pathway or NP combined with the Nampt pathway.

The importance of NAD^+^ is reflected through the activity of NAD^+^-depleting enzymes, the mediators of aging, which are mostly induced by stress factors, such as DNA damage, oxidative stress, and inflammation. The major downstream mediators are sirtuins, the NAD^+^-dependent deacetylases/deacylases. Sirtuins are conserved regulators of aging and longevity in diverse organisms, and regarded as the master switches of metabolism [22] due to their numerous regulatory functions in metabolism, DNA repair, stress response, chromatin remodeling and circadian rhythm. (Table 1) [2,23]. Together with sirtuins, PARPs use NAD^+^ to produce a chain of ADP-ribose (ADPR) molecules. PARP1 and PARP2 respond to DNA breaks in the nucleus and facilitate the process of DNA repair [23]. As DNA damage accumulates over time, the activation of PARPs increase, which in turn, lowers the activity of SIRT1 due to both substrate competition and PARP2′s ability to bind to the promoter of Sirt1 and repress its expression [24]. Furthermore, the content of the primary NADase in mammals, CD38, increases with age. This enzyme uses NAD^+^ to produce and hydrolyze the Ca^2+^-mobilizing second messenger, cADP-ribose [25,26,27]. CD38 can also degrade the NAD^+^ intermediates, NR and NMN [28,29], which further decreases the content of NAD^+^ [30]. The effect of CD38 on NAD^+^ content was demonstrated in CD38-deficient mice, whose NAD^+^ levels remain high. This preserves mitochondrial respiration and metabolic function with age [31]. Moreover, the inhibition of CD38 can increase NAD^+^ levels and improve glucose and lipid metabolism [32]. Apart from the ectoenzymes CD38 and CD157, SARM1 (sterile alpha and Toll/interleukin-1 receptor motif-containing 1) is an additional NAD^+^-depleting enzyme that uses NAD^+^ to promote axonal degeneration after injury and thereby decreases its overall content [33].

The immense involvement of NAD^+^ in all these processes suggests a significant potential for the treatment of various pathophysiological conditions with supplementation of NAD^+^ precursors. However, multiple options exist for supplementation, and the optimal precursor and dosage are still unclear with regard to the specific condition. For the baseline requirements of NAD^+^ synthesis, dietary tryptophan or less than 20 mg of niacin (NA, NAM) daily are sufficient. However, growing evidence demonstrates that substantially greater rates of NAD^+^ synthesis exhibit numerous beneficial or even therapeutic effects, which can be achieved by supplementation of its intermediates. These intermediates are found in a wide variety of foods, including meat, eggs, dairy, certain vegetables and whole wheat [35,36]. Specifically, NR is the third discovered NAD^+^ precursor that naturally occurs in milk and is already available as a nutraceutical. Oral supplementation with NR has been shown to increase NAD^+^ levels in multiple tissues, along with increased SIRT activity [10,11], improved mitochondrial function [37], and regenerative potential of stem cells [38]. Furthermore, NR is currently regarded as a favorable precursor since it has not been implicated to have serious side effects or flushing as opposed to other NAD^+^ precursors [14,39]. NR chloride has been given GRAS status (generally regarded as safe) which further supports its rapid implementation as a drug-like therapy. However, to adapt NR technologies for therapeutic use, it is necessary to determine oral availability, therapeutic dosage, and utilization in different tissues. Human digestion and the microbiome [40] additionally play important roles in NR metabolism, which has yet to be characterized in detail. In this regard, we have summarized the available data from human and animal studies on NR effects on metabolic, neurodegenerative, and cardiovascular disorders, along with the advantages of NR over other available NAD^+^ precursors. We also considered immunomodulatory effects in this review, as recent data indicates that NR can support the treatment of infections, including those caused by SARS-CoV-2 that has resulted in the recent Coronavirus pandemic. The current state of NR research including its bioavailability, safety, and effects on oxidative stress and longevity is described in this paper.

## 2. Search Strategy and Selection of the Papers

We searched mostly the PubMed database for papers published in open-access peer-reviewed journals. In addition, we used available abstracts of papers or entire articles on ResearchGate without open access. We used publication date as a priority to select the papers, and most of the reviewed papers have been published within the last 10–15 years. Older papers were considered more as a source of fundamental discoveries. Additional databases were also searched via Google using the following keywords: nicotinamide riboside (NR) bioavailability, NR safety, NR supplementation, NR effects on metabolism and insulin, NR effects on neurodegenerative disorders, NR effects on longevity, NR effects on liver health, and similar.

## 3. Effects of NR on Metabolism and Age-Associated Pathophysiology

### 3.1. Effects on Insulin Sensitivity, Liver Health and Other Metabolic Functions

Along with its intermediates, NAD**^+^** plays an essential role in metabolism, and evidence suggests that an increase in NAD^+^ levels can exhibit ameliorating effects on metabolic disorders, such as type 2 diabetes (T2D), metabolic syndrome, and nonalcoholic fatty liver disease (NAFLD) [14,41,42]. Furthermore, NR is one of the NAD^+^ intermediates that also serves as a precursor of NADH, as well as hepatic NADP**^+^** and NADPH [14]. Since the hepatic NAD**^+^** metabolome is regarded as a function of prediabetic (PD) and T2D mouse models, NADP^+^ and NADPH can be used to assess the progression of the disease. Namely, both NADP^+^ and NADPH are important for resistance to oxidative stress, while NADPH is thought to be the major contributor to insulin resistance [42]. The significantly decreased levels of hepatic NADP**^+^** and NADPH in PD and T2D mice are restored with supplementation of NR [41]. In mice models, NR can increase NAD^+^ metabolism and thereby improve glucose tolerance, reduce weight gain, and exhibit neuroprotective effects against diabetic neuropathy and liver steatosis [41]. Similarly, in mice models of high-fat-induced obesity, a dose of dietary NR as small as 400 mg/kg/day was shown to improve insulin sensitivity and protect mice from weight gain [37]. However, these results have not yet been replicated in humans, as 12-week supplementation of NR with a dose of 2000 mg/day was not able to improve insulin sensitivity and other metabolic parameters in insulin-resistant, obese men [43]. Additional research is required to determine the long-term effects of NR on insulin sensitivity [43]. On the other hand, there is evidence that NR administration in mice models increases the activity of SIRT1, an important factor in the prevention of T2D and preservation of insulin sensitivity [37,44]. Moreover, SIRT1 inhibits effects of oxidative stress in T2D mice [45], promotes glucose-stimulated insulin secretion from pancreatic β-cells [46,47], and protects against insulin resistance in peripheral tissues [48], whilst SIRT1 overexpression promotes fatty acid oxidation and inhibits lipogenesis, protecting the liver from steatosis. In addition, sirtuins are downregulated in NAFLD patients, a state characterized by liver steatosis [49]. NAFLD is widely considered a hepatic manifestation of metabolic syndrome as it is found to be associated with T2D, obesity, and insulin resistance [50]. Via activation of SIRT1 and additional factors associated with cholesterol homeostasis, NR could potentially reduce cholesterol levels and improve liver health [51,52,53,54]. Fat accumulation is also potentially reduced through a mechanism that involves the induction of the mitochondrial unfolded protein response [44]. In regenerating liver, NR reduces lipid accumulation, promotes hepatocyte replication, and increases hepatic ATP content leading to a faster regaining of liver weight in mice [55]. Moreover, dietary supplementation of NR was able to restore NAD**^+^** levels caused by impaired biosynthesis in a mouse model of hepatocellular carcinoma, and thereby prevent both DNA damage and tumorigenesis [56]. Altogether, there is sufficient evidence indicating that increasing NAD^+^ content with NR supplementation can be considered a promising therapeutic strategy for metabolic dysfunctions, including T2D and NAFLD.

### 3.2. Effects on Cardiovascular Diseases

Disruption of NAD^+^ homeostasis due to mitochondrial dysfunction is central in the development of cardiac hypertrophy and heart failure (HF) and has been reported in several models of HF including pressure overload, myocardial infarction, and angiotensin II infusion [57,58,59,60,61,62]. Furthermore, a shift from fatty acid oxidation and oxidative phosphorylation to other forms of substrate metabolism (glycolysis and ketone oxidation) often occurs in the development of HF [63,64], while the NAD^+^/NADH ratio also decreases [64]. The change in oxidative-reductive capacity further increases cardiac susceptibility to stress. The level of protein hyperacetylation also increases, driven by decreasing NAD^+^-dependent deacetylation both in mouse models of hypertrophy and in human patients with ischemic HF or dilated cardiomyopathy (DCM) [64,65]. NR supplementation normalizes the myocardial NAD^+^/NADH ratio and exhibits protective effects in adverse cardiac remodeling, while long-term supplementation increases nucleocytoplasmic protein acetylation by stimulating citrate and acetyl-CoA metabolism and antioxidant gene expression [66]. By improving NAD^+^ homeostasis and activating NMRK2, NR can further prevent the deterioration of cardiac function and adverse remodeling, which are both early and persistent events in a mouse model of DCM leading to HF.

In several models of cardiac injuries, the NAMPT enzyme is repressed [62], while Nmrk2 expression is robustly upregulated [66]. A similar shift was observed in human failing heart in models of cardiomyopathy [66]. Hence, it was proposed that the activation of the NMRK2 pathway represents a common adaptive mechanism in the failing heart, while the Nmrk2 gene can be activated in response to NAMPT inhibition. Furthermore, the shift from NAMPT to NMRK2 for NAD^+^ synthesis is an energy-sparing mechanism that may be favored, since NMN synthesis from NR by NMRK enzymes requires a single ATP, while synthesis from NAM by NAMPT requires more than three ATP equivalents (Figure 2). Although the NMRK2 pathway is activated in the HF mice, the myocardial NAD^+^ level is depressed, which suggests that circulating and tissue levels of NAD^+^ precursors are insufficient to sustain cardiac NAD^+^ synthesis on a regular rodent diet. This supports the interest in NR supplementation to improve this condition. Moreover, a strong beneficial effect of NR was discovered in mouse models of HF with the preservation of cardiac function and a limitation of cardiac remodeling, that was associated with maintained NAD^+^ levels in the heart. This further suggests that oral NR supplementation is a powerful approach to preserve cardiac function and limit remodeling in DCM.

NAD^+^ precursor supplementation also has the potential to protect against adverse cardiac remodeling by additional mechanisms of activating SIRTs (Table 2) and maintaining Ca^2+^ homeostasis [67]. Namely, in vivo activation of SIRT1 protects against cardiac hypertrophy, metabolic dysregulation, and cardiac inflammation in a mouse model of cardiac hypertrophy, and exhibits protective effects in other models of cardiac dysfunction [68,69,70,71]. In addition, SIRT2 and SIRT6 have emerged as prominent cardioprotective SIRTs [72,73], as deficiency of SIRT2 intensified cardiac hypertrophy in aged mice and mice stressed with angiotensin II [73], while a loss of SIRT6 in mice resulted in the development of cardiac hypertrophy and HF [74]. The activity of mitochondrial SIRTs is implicated in cardiac remodeling and the development of HF, including SIRT3 which appears to be required for maintaining cardiac function [75]. Data further suggests NR as a favored NAD^+^ precursor in mitochondria [76], while in vivo NR effects have been interpreted as dependent on mitochondrial sirtuin activities [37,77]. However, the importance of nucleocytosolic targets should not be excluded [1,78]. NR-induced reduction of systolic blood pressure (SBP) and aortic stiffness [39], two clinically important risk indicators of cardiovascular function and health [79,80], may occur due to nuclear and cytosolic SIRT1 activation (Table 1). Namely, NAD^+^ is an obligate substrate for the deacetylase SIRT1, which has been implicated in the maintenance of healthy vascular function [81,82,83]. Although NAD^+^ involvement in numerous physiological responses is not yet fully understood, documented health benefits in mice models validate the increasing interest in the translation of NR to therapy for cardiovascular diseases, especially in HF and cardiac hypertrophy.

### 3.3. Effects on Neurodegenerative Disorders

Neurodegenerative disorders are associated with DNA damage and oxidative stress, which accumulate with age [85] leading to impaired mitochondrial function [86]. In addition, NAD^+^ depletion was observed during the aging process in multiple animals, including humans, and is considered the major risk factor for Alzheimer’s disease (AD) [87]. When administered in AD mouse models, NR displays beneficial effects on both oxidative stress and DNA repair by increasing NAD^+^ levels [88]. Additionally, NR can improve other aspects of AD neuropathology including pTau, amyloid-β, neurogenesis, neuroinflammation, hippocampal synaptic plasticity, and cognition [89,90]. Specifically, NR treatment reduce neuroinflammation and amyloidogenesis in the whole brain of high-fat diet (HFD)-fed mice, by decreasing amyloid-β levels and several inflammatory markers (NLRP3, CASP1, IL-1, TNF-α, and IL-6) [91]. Since brain inflammation is closely related to cognitive impairment [92,93,94], cognitive function and recognition memory could be attenuated by NR treatment in only 6 weeks [91]. Furthermore, increased PARylation, another hallmark of AD, could be decreased in AD mice with NR supplementation [90]. Increased PARylation has also been detected in several other neurodegenerative disorders that involve DNA repair defects, including Cockayne syndrome, xeroderma pigmentosum and ataxia-telangiectasia. Nevertheless, PARP-mediated NAD^+^ depletion was recently confirmed to play a major role in the pathogenesis of these disorders. Despite the underlying DNA repair deficiencies, NR could dramatically improve the phenotype of each of these conditions in mouse models, while prolonging survival by more than three times in ataxic mice [95,96].

Another early event that occurs in acute cerebral injury and in chronic neurodegenerative diseases, including Alzheimer’s and Parkinson’s diseases, is axonal degeneration [97,98,99,100]. In such cases, axonal degeneration is caused by excitotoxicity, which is another feature implicated in most neurodegenerative disorders that affect the central nervous system. Interestingly, a strong NAD^+^ depletion in neurons has been revealed during excitotoxicity [101,102,103,104], whereas mice injected with NR were protected from excitotoxicity-induced axonal degeneration [105]. Among three tested NAD^+^ precursors (NA, NAM, and NR) including NAD^+^, only NR could prevent axonal degeneration by altering the local NR metabolism within the axon [105,106]. Namely, NR prevented nuclear condensation and axonal degeneration in neurons of Nmrk2-KO mice, by inducing Nmrk1 [105], which suggests that Nmrk1 might be the key mediator of the neuroprotective activity of NR. This neuroprotective effect depends on both mitochondrial and axonal NAD^+^ content [78]. At present, two possible mechanisms of neuroprotection have been proposed: increasing mitochondrial NAD^+^ to support SIRT3 [77] and preserving axonal NAD^+^ to supply damage-induced SARM1 activation [107]. Via SIRT3 activation, a NR-induced increase in NAD^+^ levels can have additional beneficial and possible therapeutic effects. Specifically, NR has been shown to prevent noise-induced hearing loss and neurite retraction from hair cells in the inner ear through a SIRT3-dependent mechanism [77]. Moreover, SIRT3 and 5 are both critical for the health of retinal photoreceptors [108]. SIRT3 activity is sensitive to the reduction of NAD^+^ content that has been detected in multiple disorders with retinal degeneration, including age-associated dysfunction, diabetic retinopathy, and light-induced degeneration in mice [108]. This suggests a potential therapeutic treatment with NR for a variety of disorders that include photoreceptor degeneration.

Furthermore, a general decrease in NAD^+^ levels has been observed in neuromuscular diseases which are often caused by inherited mutations that lead to progressive skeletal muscle weakness and degeneration [38]. Increasing NAD^+^ content with NR can stimulate energy production and improve mitochondria function.NR has been shown to have therapeutic effects in several muscle disorders in mouse models. Although supplementation with NR could not correct the underlying genetic defects, it could improve mitochondrial abundance and function in two different mitochondrial myopathies [109,110]. Furthermore, NR could reverse the progressive wasting syndrome in skeletal muscle in mice lacking Nampt whilst restoring endurance, in as little as 1 week of treatment [111]. Accordingly, NR was found to confer increased endurance and improved cold tolerance in the HFD-fed mice. However, whether NR has significant benefits in lean, healthy muscle is less clear, as only a nonsignificant trend toward increased endurance was observed in regular chow-fed mice [37].

Interestingly, decreased NAD^+^ levels and Nampt expression were also observed in Duchenne’s muscular dystrophy (DMD), a condition histologically and transcriptionally similar to progressive wasting syndrome [112,113]. DMD is also characterized by increased PARP activity, fibrosis, and muscular degeneration. NR was found to improve muscular function and heart pathology in mdx mice models of DMD and decrease PARylation, inflammation, and fibrosis [112]. Moreover, NR treatment has been shown to improve stem cell function and thereby ameliorate the muscle wasting phenotype in mdx mice, supporting the use of NR for the human condition [38,112]. Improvement in stem cell function appears to be a general phenomenon during NR treatment and has been suggested to underlie a small, but significant extension of lifespan in mice [38]. Altogether, these findings support that NR could be effective in managing the progression of muscular dystrophy and degeneration, by improving muscular strength, rejuvenating senescent muscle stem cells, and reducing levels of inflammation and fibrosis.

### 3.4. Effects on Longevity

Calorie restriction (CR) is considered the most effective approach to extend lifespan in eukaryotes since the first report of lifespan extension in wild-type yeast cells via regulation of Sir2 and NAD^+^ [114]. CR life-prolonging effects may partly be mediated via increased sirtuin function, while the requirement of NAD^+^ for their activity suggests a possible connection between aging and metabolism. However, the nutritional approach for increasing Sir2 activity and longevity has been accomplished by engineered gene overexpression in yeast [115], while NA failed to extend lifespan, and NAM shortened it [116,117]. On the other hand, as with CR, NR can increase NAD^+^ levels and Sir2 function, while exogenous NR promotes Sir2-dependent repression of recombination, improves gene silencing, and extends lifespan without calorie restriction [11]. Moreover, the mechanism of action of NR is completely dependent on increased net NAD^+^ synthesis through the Nrk1 and the Urh1/Pnp1/4 pathways. The latter is Nrk1 independent and represents a newly discovered NR salvage pathway [11]. Furthermore, a study in mouse models documented that a one-day fast increases NAD^+^ in the liver [118] whereas CR elevates NAD^+^ and reduces NAM in the brain [119]. This implies that the increased levels of NAD^+^ appear to mediate several beneficial effects of CR, supporting the life-prolonging effects of NR supplementation. These effects are mediated via improvement of metabolism and decrease in chronic inflammation, a hallmark of aging [35]. Preclinical studies have reported that NR reduces macrophage infiltration in damaged muscles [38,112] and attenuates plasma TNF-α in models of fatty liver disease [44]. Nevertheless, a recent clinical study confirmed NR availability in muscular tissue in aged human subjects [120] and its anti-inflammatory effects. Namely, a 21-day supplementation of NR decreased numerous circulating inflammatory cytokines [120], implying additional mechanisms through which NR can potentially modulate the aging process and thereby exhibit life-prolonging effects. While the exact mechanisms through which NR exerts these effects remain unclear, the apparent health benefits described indicate positive effects of NR on longevity.

## 4. Infection Treatment and Immunomodulatory Effects

NAD^+^ intermediates have been recognized for their beneficial health effects during infection with several pathogens. Studies have confirmed antimycobacterial effect of NAM in patients infected with *Mycobacterium tuberculosis* [121,122], while immune-mediated elimination was reported for *Staphylococcus aureus*, including MRSA and other major human pathogens such as *Klebsiella pneumoniae* and *Pseudomonas aeruginosa* [123]. In addition, NAM and its analogues exhibited antiviral effect in patients with HIV [121] and hepatitis B [124]. The potential treatment with NAD^+^ intermediates has been recognized recently for combating COVID-19 infection that currently lacks efficient therapeutic or preventive agents and represents a global concern for public health.

SARS-CoV-2 infection triggers a maladaptive immune response. Notably an exaggerated proinflammatory response leading to a “cytokine storm” in the lung tissue, and lymphopenia with a drastic decline of CD4+ and CD8+ T cells [125]. On the molecular level, as the innate immune response activates to fight the infection, the activation of PARPs increases due to extensive DNA damage and IFN-induced MARylation (mono-ADP-ribosylation) of the target SARS-CoV-2 proteins [126,127]. PARPs response is required for inhibition of viral replication [128]; however, this antiviral effect is reversed by the ADP-ribosylhydrolase macrodomain of the viral nonstructural protein, nsp3, whose activity is required for virulence [126,127,129]. In addition, nsp10 of SARS-CoV was found to inhibit electron transport at the NADH site of complex I in the mitochondrial electron transport chain [130], suggesting that key events in the innate immune response to viral infections are occurring within the infected cell’s NAD^+^ metabolome [131]. Recent study investigated PARPs expression and NAD^+^ metabolome dysregulation due to coronavirus infection. The examined SARS-CoV-2 infected cell lines of a ferret and deceased patients’ lungs showed disturbed NAD**^+^** metabolism and gene expression with respect to synthesis and utilization of NAD**^+^** [131]. Furthermore, the expression of the NMRK1 pathway was upregulated together with the expression of the concentrative nucleoside transporter CNT3, indicating a higher capacity for NR conversion to NAD^+^ and NADP^+^ during the infection [10]. An upregulation of the NMRK gene was previously associated with therapeutic efficacy of NR [66,105]. In addition, the expression of NNMT (nicotinamide N-methyltransferase) was decreased [131] due to lower NAM methylation, suggesting a promotion of the NAM salvage pathway [132] and increased efficiency of NR treatment to replenish NAD^+^ [131]. These data indicate that boosting NAD^+^ content through NAM and NR kinase pathways may restore antiviral PARPs functions to support innate immunity to SARS-CoV-2 [131].

Upon activation of the adaptive immune response, the overexpression of CD38 in both CD4+ and CD8+ lymphocytes further exaggerates NAD**^+^** depletion [133,134] leading to increased production and release of proinflammatory cytokines, reactive oxygen species, and macrophage infiltration [135,136]. Furthermore, drastic NAD^+^ depletion impairs the function of sirtuins, the regulators of cell death and viability [133]. Specifically, SIRT1 regulates the expression of genes including tumor suppressors, cytokines and proto-oncogenes and ultimately modulates inflammation, cell survival, and apoptosis mechanisms [137]. Loss of sirtuin function along with increased oxidative damage and an overall energy decrease finally culminates in cell death. Replenishment of NAD**^+^** body content could restore energy levels and impaired sirtuin function and possibly rebalance the maladaptive immune response to SARS-CoV-2 infection. Namely, both SARS-CoV and SARS-CoV-2 induce maladaptive hyperinflammation followed by increased leucocyte infiltration into the lungs resulting in extensive tissue damage and subsequent organ failure with reduced lung capacity [138,139,140]. Emerging evidence has demonstrated that NAD**^+^** is released during the early phase of inflammation and has an immunoregulatory role in vivo [141,142]. Moreover, niacin was previously suggested as an anti-inflammatory therapy in one preclinical study as a potent agent to decrease proinflammatory cytokines, including IL-1, IL-6, and TNFα [143]. NR can similarly decrease IL-2, IL-5, IL-6, and TNFα [120]. Targeting IL-6 has been recently proposed as a promising treatment to block the inflammatory storm, especially in severe COVID-19 patients [132]. Furthermore, niacin might also decrease neutrophil infiltration, while exhibiting a prolonged anti-inflammatory effect during ventilator-induced lung injury. However, niacin exacerbated hypoxemia regardless of the neutrophil infiltration decrease, suggesting a different cause of hypoxemia, independent of neutrophil decline [144,145], which requires further investigation. In addition, high efficiency of vitamin B3 (niacin or nicotinamide) in preventing lung tissue damage was confirmed in several animal models with bleomycin- and LPS-induced lung injury [146,147,148]. Considering vitamin B3′s strong lung-protective effects, it has been proposed as an early treatment-supporting agent against COVID-19 [146]. This implies that NR should be considered as a potential therapeutic or supporting agent to reduce hyperinflammation and regenerate damaged lung tissue.

## 5. Bioavailability and Safety

The bioavailability of NR can be tested by measuring NAD**^+^** levels or other relevant biomarkers such as nicotinic acid adenine dinucleotide (NAAD) in the cells of the target tissue or in the blood. Throughout numerous observations in a wide variety of mammalian cell lines including liver, skeletal muscles, and brown adipose tissue, NR was documented to enhance NAD^+^ levels [37]. Conversely, the NAD^+^ levels did not significantly increase in the brain or white adipose tissue [37]. It was suggested that the observed differences are caused due to differential Nmrk expression in the specific tissues. While Nmrk1 is expressed ubiquitously, Nmrk2 is mainly expressed in cardiac and skeletal muscles, but also detectable in the liver and brown adipose tissue which might explain the better ability of these tissues to respond to NR. On the other hand, NR is very unstable in the blood, which makes it difficult to measure and detect. Despite its instability, the development of reliable methods for collection, processing, and measuring has enabled the determination of the pharmacokinetic profile of orally administered NR. The study conducted in both healthy human volunteers and mice reported that an NR dose of 1000 mg twice daily (2000 mg in total) can significantly increase steady-state, whole-blood levels of NAD^+^ (up to 2.7 fold after one dose) [149] and effectively stimulate NAD^+^ metabolism [39,149,150]. The studies also confirmed that measurable, biological effects on NAD^+^ levels can be achieved by chronic oral NR supplementation with no serious adverse effects [39,150]. Specifically, there were no severe side effects reported such as flushing, pruritus, hyperglycemia, hyperuricemia, or increased enzyme activity in the liver or muscle [149,150,151,152]. However, the blood NAD^+^ response did not appear to correlate with the absorption pattern of NR and the peak in NAD^+^ increase was reached after 9 days [150]. Furthermore, it was suggested that repeated dosing would be required to prevent wide fluctuations in body levels of NR due to a relatively short elimination half-life of NR observed in several subjects; however, continuous blood levels of NAD^+^ suggest that twice-daily or even once-daily dosing of NR may be sufficient to achieve a desired clinical outcome [150].

On the other hand, the apparent oral bioavailability of a 1000 mg dose of NR was highly variable among individuals [150]. The instability of NR in blood samples observed across several studies [150,153] could be one contributing factor, although it cannot completely explain the observed variability. Another proposed explanation was the NR hydrophilicity [15] since NR is expected to exhibit low passive permeability across the human intestinal mucosa [150]. Additionally, the interindividual variation in the transport mechanism of NR in the intestinal system might also affect the oral absorption of NR. Furthermore, it was proposed that NR can be degraded to NAM in the gut, whereas, another study showed that NR is metabolized to NAM in the liver and might explain low bioavailability in other tissues [154]. Subsequently, NAM can be absorbed and converted to NMN, and further metabolized to NAD^+^ or dephosphorylated to NR. In this case, the degradation of NR to NAM in the gut, which presumably involves purine nucleoside phosphorylase in mammalian and bacterial cells, may be the variable step involved in the oral intake of NR [155]. Furthermore, multiple pathways for the conversion of NR to NAD^+^ were identified in a study with male human subjects and C57Bl6/J mice [149]. Interestingly, as a response to NR, a remarkable increase (45-fold) in NAAD was reported [149] indicating another possible conversion pathway of the NR to NAD^+^. These studies suggest that causes in the variable oral bioavailability might be revealed with further investigation of NR metabolism and transport.

## 6. Advantages Compared to Other NAD^+^ Precursors

At the current time, NR is emerging as a leading candidate due to its bioavailability, safety, and strong ability to raise NAD^+^ content compared to other precursors [149]. Among diverse NAD^+^ precursors, NMN and NR presented better pharmacokinetic and pharmacological properties [156]. The bioavailability between the NAD^+^ precursors (NMN, NR, NAM, and NA) was assessed in preclinical studies as the ability to elevate intracellular NAD^+^. NR was able to increase NAD**^+^** levels in the liver of mice, exhibiting greater oral bioavailability than NAM, which was, in turn, more orally bioavailable than NA [149]. In addition, animal studies have reported that equimolar oral NR is superior to NA and NAM in elevating NAD**^+^** content in the liver [149]. Similarly, the NAD^+^ content in muscles could be significantly increased with NR and NA, but not with NMN [149]. The three precursors (NA, NMN, and NR) differed in the degree to which they promote the accumulation of ADPR, the measure of sirtuins activity, and other NAD^+^-consuming activities [149]. Namely, NR was found to increase ADPR ~3 more than NAM, which supports NR as a favored NAD^+^ precursor to increase NAD^+^ and NAD^+^-consuming activities in the liver [149]. Moreover, the activity of sirtuins was stimulated after NR-induced NAD^+^ levels increased [37]. Both SIRT1 and SIRT3 activities increased in vitro and in vivo [37], which favors the hypothesis that NR can increase NAD^+^ levels in at least mitochondrial and nuclear compartments. NR’s ability to increase NAD^+^ in different subcellular compartments represents the crucial difference compared to other approaches of increasing intracellular NAD**^+^** levels. Although all three precursors, NA, NAM, and NR can raise both NAD^+^ and NADP^+^ levels [14,149,157], they all exhibit distinct physiological responses. For example, NA shows lowering effects on blood lipid levels, and it is used to treat dyslipidemia [151]. However, NA is implicated with flushing at doses higher than 50 mg/day [151]. On the contrary, NAM does not affect lipid blood levels, yet it can exhibit sirtuin-inhibiting effects at higher doses [116,149]. Among the three mentioned precursors, only NR could prolong survival and induce hematopoietic stem cell regeneration, as documented in a study with mice treated with irradiation [158]. Furthermore, orally administered NR was found to improve resistance to and reversal of chemotherapeutic neuropathy [159]. This implies advantages of the NR precursor for potential use in cancer patients undergoing chemo- or radiotherapy. The bioavailability of NAD^+^ precursors can be assessed by measuring NAAD levels. Namely, NAAD represents the most sensitive biomarker of effective NAD^+^ supplementation since it is undetectable in the blood prior to supplementation, and the increase of its levels has been observed in the liver after orally administered NAD**^+^** precursors. NR was found to increase NAAD by at least 45-fold compared to baseline [149]. In addition, NR could significantly elevate NAAD heart content, where the increase of NAD^+^ occurs in the absence of a steady-state NAD^+^ increase [149]. Surprisingly NA, the only precursor expected to convert to NAD^+^ through a NAAD intermediate, produced the least NAAD, while NAM and NR both produced peaks of hepatic NAAD [149].

High availability of NR has been observed in the normal human diet. NR does not require conversion to enter the cell, which could partially explain the high level of availability. On the contrary, NAD^+^ and its precursors have to be converted to either NR or NAM before entering the cell [153]. While NAD^+^ and NMN are being converted to NR extracellularly by CD73 [28], their cellular conversion depends on the NMRK pathway [153,160]. However, a NMN specific transporter in the gut encoded by Slc12a8 gene, has recently been identified by Grozio et al. [161]. Hence, the utilization of NR and extracellular NAD^+^ are limited by the activity of the NMRK pathway [153]. Conversely, another candidate has recently emerged that can increase NAD^+^ levels through NMRK independent pathway. Namely, one study reported that NR reduced form (NRH) is bioavailable in mice and indicated its great potential for therapeutic application [162]. Since NR is unstable in blood circulation, partially due to degradation to NAM [153], its ability to reach the peripheral tissues after oral administration has been compromised [162]. This limitation could be overcome by the administration of NRH as it appears to be more stable than NR and does not undergo direct degradation in plasma [162]. NRH is detectable in circulation after oral intake or intraperitoneal injection, and it was found to increase NAD**^+^** in both cultured cells (5–10-fold above the baseline) [162,163] and mice, in a more potent and faster manner than NR. Moreover, a NMRK-independent pathway for the NRH-induced NAD**^+^** increase has been demonstrated [162], which is consistent with a recent discovery of a novel biosynthetic route of NAD^+^ from NRH through the NMNH intermediate, where adenosine kinase (ADK) acts as a NRH kinase [164]. This study also confirmed the presence of endogenous NRH in the liver of mouse models establishing NRH as a valid natural precursor of NAD^+^.

On the other hand, NR may be a more suitable NAD**^+^** precursor regarding the side effects. Although NA and NAM can enter the NAD^+^ salvage pathway, several preclinical studies have confirmed that both NA and NAM can cause painful flushing sensations at therapeutic doses or other toxic effects [14,165,166]. Although NMN exhibits significant beneficial pharmacological activities in preclinical studies, there is still a lack of sufficient clinical and toxicological data. To date, there are no available tests of NMN safety and human oral availability although recently, one clinical study with ten healthy men confirmed that a single oral dose ranging from 100–500 mg is safe and effective, with no significant deleterious effects [167]. On the other hand, NR was confirmed across a number of studies as well-tolerated, up to 2 g of a daily dose, and it was not found to be associated with flushing or any severe side-effects [39]. Specifically, NR administration can elevate NAD**^+^** levels in mammalian cells and tissues without activating GPR109A that mediates nicotinic acid-induced flushing [168].

## 7. NR Derivatives and Supplementation

During the state of physiological equilibrium, biosynthetic pathways rely on dietary sources of tryptophan (Trp), while NAD**^+^** precursor vitamins are compensating during periods of NAD**^+^** depletion. However, dietary precursors may become insufficient to maintain the NAD**^+^** levels in pathological conditions [14,41,169], thereby emphasizing the necessity of NAD**^+^** precursor supplementation. Besides the well-known vitamin B3 supplements (NA and NAM), NR and its phosphorylated form (NMN) have only recently become orally available as precursors of NAD**^+^** [11,150,170,171]. On the other hand, the synthesis and manipulation of NR remain challenging, especially regarding its relatively labile glycosidic bond and the instability of NR salts [172]. However, great potential for the development of novel drugs and structural analogs of existing drugs can be achieved with the introduction of various chemical groups on the backbone of the molecule [172]. In general, there are two reported categories of synthetic pathway for nicotinamide riboside salts (NR^+^X^−^). Since only the β-form of NR**^+^** has biochemical and medical relevance, a valuable method of NR^+^ synthesis should provide high levels of β-stereoselectivity. From two main synthetic pathways of NR, only one is predominately exploited and developed in terms of synthetic efficiency, stereoselectivity, and overall yield [172]. This method embodies the reaction between NAM or its analogs or derivatives and a peracylated (halo)-d-ribofuranose, resulting in the acylated intermediate that is subsequently converted into the desired NR^+^X^−^ [173,174]. The synthetic glycosylation conditions depend on the nature of the sugar component [172]. These conditions differ whether 1-halo-2,3,5- tri-o-acyl- or 1,2,3,5-tetra-*O*-acyl-d-ribofuranose is used since fully acylated ribofuranoses require the use of Friedel–Crafts catalysts that need to be activated as glycosylation reagents [172].

Furthermore, the X-ray structures of NR salts have already been determined. The NR derivatives nicotinamide-β-d-riboside chloride, nicotinamide-β-d-riboside bromide, thionicotinamide-β-d-riboside bromide, nicotinamide-β-d-riboside triacetate bromide, and thionicotinamide-β-d-riboside triacetate bromide were successfully crystallized by the vapor diffusion method [175]. Moreover, it was found that a crystalline form of nicotinamide ribose chloride has advantageous properties compared to amorphous forms, considering the possibilities for better purification [176]. However, further development of NR chemical synthesis along with a better understanding of the chemical versatility and reactivity of the ribosylated forms on the nicotinoyl moiety would offer more reliable, more scalable, and more reproducible preparations of NR**^+^**X**^−^** salts [172]. In addition, effective modifications of the riboside residue of NR^+^ at the 5′-hydroxy position could ensure higher yields, better recovery, and improved purification strategies [172]. This will enable the improved preparation of new pharmaceutically acceptable forms and potentially therapeutically useful forms of NR^+^ in addition to the atom-efficient syntheses of isotopically labeled NR^+^ analogs and derivatives [172].

Regarding a high demand for NR use in studies and for supplementation, new and reliable synthetic methods for NR production have been developed [177] during the past few years, enabling larger quantities available for cell-based studies and animal feeding experiments [37,88]. NR has become available as a supplement in a form of crystalline chloride salt, in July 2013, with the brand name NIAGEN (Chromadex Inc., Irvine, CA, USA). In a 90-day toxicology rat study, the crystalline form of NR chloride was tested and the lowest observed adverse effect level (LOAEL) was 1000 mg/kg/day, whereas the no observed adverse effect level (NOAEL) was 300 mg/kg/day [178]. NR has also been tested in six clinical trials [39,43,149,150,179,180], where NR was established as safe for short (8 days) [150] and long-term (6 weeks) [39,179] use along with confirmed oral availability [149]. Furthermore, a randomized 8-week placebo-controlled trial with three different doses (up to 1 g) of NR, tested in overweight and healthy adults, reported that NR chloride is safe and orally available [178].

## 8. Conclusions

Remarkable discoveries regarding numerous beneficial health effects of NR in preclinical studies might finally culminate in a breakthrough, and enable treatment of a large number of metabolic and neurodegenerative disorders. NR effects are currently being investigated in a significant number of clinical trials [181], including research into diverse cardiovascular diseases, neural and cognitive functions, metabolic disturbances, muscular and kidney injuries, aging, chemotherapy. In addition, fundamental research on NR transport and metabolic pathways will further support a rapid translation to effective therapeutic use. The advantages of using NR over alternative NAD^+^ precursors, including its safety and efficiency, suggest the possible replacement of niacin as a general supplement in the near future. However, the use of NR as a nutritional supplement still has certain limitations with respect to its production methods, including low yield, the use of expensive or hazardous reagents, and pharmaceutically unacceptable species that may be toxic or biologically intolerable by other means. With the rising number of patents, these limitations are being overcome with the development of new or improved methods for chemical synthesis of NR and its derivatives. This will enable robust, cost-effective production with higher purity and stereoselectivity. Extensive research into NR might also lead to its global availability in supplementation use and novel therapeutic strategies, most importantly for pathophysiological conditions that currently lack efficient treatment.

## Figures and Tables

**Table 1:** Central role of sirtuins in DNA repair, cell cycle control and mitochondria function (adapted from Meng et al. [34]).

	Localization	General Function	Function
SIRT1	Nucleus, Cytosol	DNA repair	Glucose metabolism, differentiation, insulin secretion, neuroprotection, vascular protection
SIRT2	Cytosol, Nucleus	Cell cycle	Adipose tissue development and functionality, blood glucose homeostasis, modulation of peripheral myelination
SIRT3	Mitochondria, Nucleus, Cytosol	Mitochondrial metabolism	ATP homeostasis, ROS detoxification, tumor suppression, DNA repair, neuroprotection, apoptosis suppression
SIRT4	Mitochondria	Mitochondrial metabolism	Insulin secretion, DNA repair, apoptosis suppression
SIRT5	Mitochondria, Cytosol, Nucleus	Mitochondrial metabolism	Urea cycle, ketone body formation, nitrogenous waste management, ROS detoxification
SIRT6	Nucleus (Chromatin)	DNA repair	Telomerase protection, genome stability, cholesterol homeostasis, glycolysis and gluconeogenesis
SIRT7	Nucleus (Nucleolus)	rRNA transcription, cell cycle	Cardiac protection

**Table 2:** The roles of sirtuins in heart failure development acquired from experiments on knock-out and transgenic mice (adapted from Pillai et al., [84]).

SIRT1	Heart cell growth and development, mediation of cardiac hypertrophy, protection from ischemic injury; partial deficiency protects from pressure overload-induced hypertrophy and failure
SIRT2	Mediating ischemic injury due to attenuated programmed apoptosis
SIRT3	Protection from age-induced hypertrophy, fibrosis and contractile dysfunction, prevents susceptibility to cardiac hypertrophic stimuli
SIRT6	Protection from cardiac hypertrophy and heart failure
SIRT7	Protection from spontaneous cardiac hypertrophy and inflammatory cardiomyopathy
SIRT1	Low to moderate overexpression attenuates age-dependent decline in cardiac functions in mice, while high overexpression induces cardiac hypertrophy and heart failure
SIRT3	Cardiac-specific overexpression protects the heart from hypertrophic stimuli by preserving mitochondrial function
SIRT6	Cardiac-specific overexpression protects the heart from hypertrophic stimuli by blocking activation of Akt signaling at the level of chromatin
